# Current Diagnostic Methods and Non-Coding RNAs as Possible Biomarkers in Huntington’s Disease

**DOI:** 10.3390/genes13112017

**Published:** 2022-11-03

**Authors:** Miguel Pellegrini, Guendalina Bergonzoni, Federica Perrone, Ferdinando Squitieri, Marta Biagioli

**Affiliations:** 1 Department of Cellular, Computational and Integrative Biology, University of Trento, Via Sommarive 9, 38123 Trento, Italy; 2 Huntington and Rare Diseases Unit, IRCCS Casa Sollievo Della Sofferenza Research Hospital, Viale Cappuccini, 71013 San Giovanni Rotondo, Italy

**Keywords:** Huntington’s disease, neurodegeneration, biomarkers, biofluids, non-coding RNA, microRNA, circRNA

## Abstract

Whether as a cause or a symptom, RNA transcription is recurrently altered in pathologic conditions. This is also true for non-coding RNAs, with regulatory functions in a variety of processes such as differentiation, cell identity and metabolism. In line with their increasingly recognized roles in cellular pathways, RNAs are also currently evaluated as possible disease biomarkers. They could be informative not only to follow disease progression and assess treatment efficacy in clinics, but also to aid in the development of new therapeutic approaches. This is especially important for neurological and genetic disorders, where the administration of appropriate treatment during the disease prodromal stage could significantly delay, if not halt, disease progression. In this review we focus on the current status of biomarkers in Huntington’s Disease (HD), a fatal hereditary and degenerative disease condition. First, we revise the sources and type of wet biomarkers currently in use. Then, we explore the feasibility of different RNA types (miRNA, ncRNA, circRNA) as possible biomarker candidates, discussing potential advantages, disadvantages, sources of origin and the ongoing investigations on this topic.

## 1. Biomarkers: A General Introduction

The emerging challenge that current clinical trials face is how a response to an experimental treatment should be assessed early in the trial, especially considering that the main affected tissue responsible for the pathology might be the brain. In therapeutic clinical trials that aim to evaluate the efficacy of potential disease-modifying treatments, biomarkers serve as valuable outcome measures [1]. Biomarkers are defined as “almost any measurement reflecting an interaction between a biological system and a potential hazard, which may be chemical, physical, or biological”, and they refer to a “measured response that may be functional and physiological, biochemical at the cellular level, or a molecular interaction” (defined by WHO [2,3]). Predictive biomarkers, reliably and objectively responding to treatment in a predictable manner, can be used to determine the effectiveness of a therapy and how a patient will respond to it. On the other hand, prognostic biomarkers, instead, are used as indicators of disease severity, ideally reflecting the underlying disease pathogenesis and linearly tracking clinical progression of the disease throughout its duration (including during the premanifest stage).

In principle, all biomarkers should be affordable and easily accessible, meaning it should be possible to repeat their measurement multiple times without having to subject the patient to particularly invasive procedures. Biomarkers should also be as specific as possible, i.e., unaffected by comorbidities and with limited variability in the general population. Finally, biomarker sampling and testing should be standardized to minimize variation between facilities.

## 2. Huntington Disease: An Opportunity to Seek Early Predictive Biomarkers of Neuro-Dysfunction and Degeneration

Huntington’s Disease (HD) is a fatal, monogenic, autosomal dominant, neurodegenerative disease, which is caused by the expansion of the CAG trinucleotide repeat within exon 1 of the HD gene (*HTT*) gene. Different individuals may display variable expansion length, with a repeat number of 35 or more considered pathogenic [4,5]. However, the range of 36–39 repeats confers reduced penetrance, where the disease might manifest later in life [6]. Longer repeats cause earlier onset, and above 60 are highly penetrant and associated with pediatric age of onset [7]. HD is endemic to all populations, but its prevalence is higher among individuals of European ancestry, where it affects about 12 per 100,000 individuals [8]. Nowadays, HD patients can unequivocally be identified via genetic testing for this dominant trait [9,10,11]. Thus, individuals with a known family history and individuals carrying the mutation while still asymptomatic, can be easily identified before developing overt clinical features of the disease [12]. These are the ‘premanifest’ individuals, those who would potentially benefit more from neuroprotective therapies, which possibly would delay the development of the neurological disease manifestations and related functional disabilities [13].

Although the causative mutation to HD was discovered almost 30 years ago, no definitive therapy to halt or delay the disease is currently available. Current pharmacological therapies are limited to the treatment of disease symptoms [14], but death will inevitably occur in 15/18 years after manifestation of the symptoms. Current investigations are exploring new therapies aimed at reducing the expression of the mutant gene and/or protein. The initial clinical trials, exploiting antisense oligonucleotides (ASOs), short, single-strand DNA/RNA sequences that cause the decay of a specific, target mRNA [15], were recently stopped because of lack of effectiveness (phase 3 trial, patient follow up for 69 weeks [16]). Nonetheless, other promising *HTT*-targeting molecular tools are emerging. For example, branaplam, originally developed as spinal muscular atrophy therapy, was recently discovered to induce the splice-in of a pseudoexon in *Htt* mRNA, destabilizing the transcript, inducing its degradation, and improving the motor performance in *Htt* mouse models [17].

Currently, the most commonly employed method to assess treatment response and monitor disease progression in therapeutic trials in HD is the United Huntington’s Disease Rating Scale (UHDRS). The UHDRS is a collection of scales that were designed to detect clinical changes in the manifestation of HD by assessing the clinical performance of HD patients in four different areas: motor and cognitive function, behavioral abnormalities and functional capacity. Therefore, until now, HD patients have been categorized primarily based on their clinical symptoms. As such, the UHDRS may not be sensitive enough to detect the subtle features seen in some premanifest individuals, particularly those who are several years away from developing the disease [18]. The main issue of this scale is its reliance on subjectiveness: in HD patients, motor function cannot be always consistently measured, as patients and operators can be influenced by external factors, such as stress, that might affect their performance. The designation of disease onset can also be arbitrary, given that the lengthy prodrome is characterized by various subtle motor and cognitive abnormalities, developing insidiously over several years. The use of disease onset as an endpoint in clinical trials would therefore require a large premanifest study population. However, an updated system, i.e., the Integrated Staging System (HD-ISS), has recently been introduced, which combines information from imaging with clinical signs and decline in daily function and better tracks disease progression, including pre-symptomatic and premanifest stages [19].

Thus, there is an increasing need in clinical research to develop and validate biomarkers able not only to assess target engagement in patients but also to track disease progression during all the disease stages, enabling better patient stratification for clinical trials.

## 3. Current Biomarkers in Huntington’s Disease

### 3.1. Dry Markers: Clinical and Imaging

In recent years, many alternative molecules and behavioral measurements have been taken into consideration as potential biomarkers for HD. Some researchers have proposed the use of clinical markers, continuous measures of motor abnormalities that can be objectively quantified, thereby improving accuracy and reducing variability. One example would be digitomotography, where a modular force transducer arrangement is used to quantify finger tapping precision, which will worsen as the disease progresses [20,21,22,23,24]. Cognitive impairment can also be assessed from premanifest stages. In this instance, the Symbol Digit Modalities test is able to assess visual attention and psychomotor speed [21,22]. However, such clinical markers did not demonstrate sufficient sensitivity to subtle changes over time during the premanifest period. On the contrary, the use of brain structural imaging has proven to be a more robust marker during the premanifest stage of the disease and in HD trials [22,23,24,25]. However, the use of structural brain atrophy as an efficacy measure can be limited because it typically occurs at a slow rate, making it rather impractical for most clinical trials. More interesting forms of visualization are functional and metabolic imaging. Thanks to these techniques, Squitieri and colleagues, observed a reduced metabolic F-18-Deoxyglucose or Fluorodeoxyglucose (FDG) uptake in the parietal lobe of riluzole-treated HD subjects compared to those exposed to placebo [26]. The metabolic measure linearly correlated with worsening of motor scores and behavioral measures.

### 3.2. Wet Biomarkers: Mutant Huntingtin and Neurofilament Light Protein

In the context of neurodegenerative diseases, such as HD, the identification of wet biomarkers is particularly important, since the affected regions (CNS and PNS) are difficult to monitor without using invasive or expensive procedures. Indeed, accessible and reliable biomarkers allow the fine tracking of the disease progression and drug benefits, without directly accessing in the brain or in the spinal cord.

#### 3.2.1. Mutant Huntingtin

**Figure 1:**
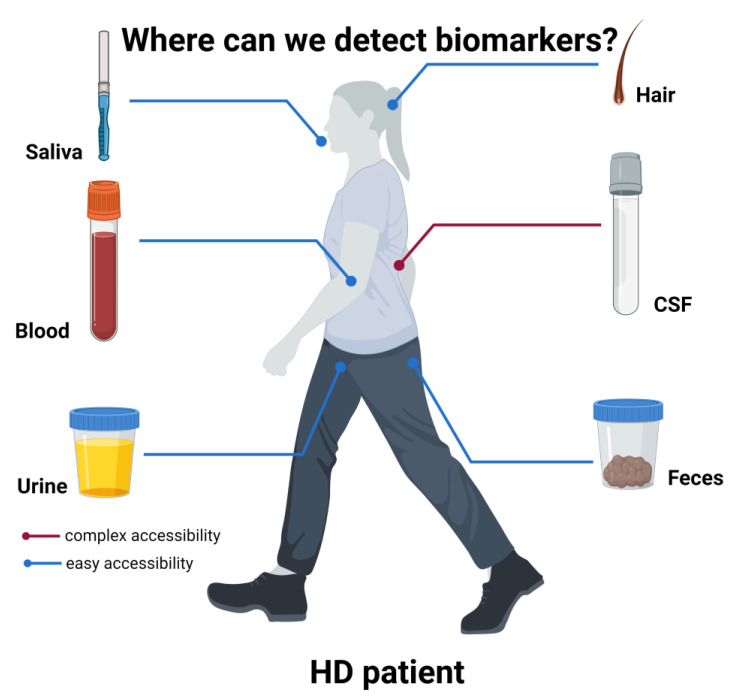
Biomarkers sources in HD patients.

The most prominent source of wet biomarkers is the Cerebrospinal Fluid (CSF) (Figure 1). In fact, one of the key functions of the CSF is the collection of waste material and metabolites coming from the CNS, thus, its composition and content can reflect the status of the brain parenchyma, especially in pathologic conditions. In HD, the CSF has been used for the quantification of the mutant huntingtin protein, currently the most commonly used biomarker for the disease [27,28], especially in huntingtin lowering therapies [29]. Differences in the mean mutant huntingtin levels were detected between premanifest and early-stage HD, but not between early-stage and moderate-stage subjects [28,30]. Importantly, mutant huntingtin levels in the CSF correlate with motor and cognitive features in premanifest and early-mid HD, but not in late HD [28,31]. Overall, mutant huntingtin has proven to be a valid prognostic biomarker, as well as a predictive biomarker for measuring treatment efficacy [32,33]. However, mutant huntingtin has a critical limitation, related to its accessibility: in fact, while this mutant protein from a ubiquitously expressed gene can be found in the peripheral blood from patients, its presence in accessible biofluids cannot be uniquely attributed to the brain, the affected area in this pathology. Thus, the CSF represents the only option that allows for the reliable measurement of brain-derived mutant huntingtin. Unfortunately, however, the CSF collection requires patients to undergo the rather invasive procedure of lumbar punctures (LP) which might cause some, rarely severe, side-effects (infections, spinal and subdural cerebral hematoma and cerebral venous thrombosis) [34,35].

A schematic representation of the various body derivatives to be evaluated as possible biomarkers is presented. While CSF is the most prominent source of biomarkers, like mutant huntingtin [27,32,36], its collection requires quite invasive procedures. Conversely, other more accessible body products have been used as source of possible biomarkers. Among them, because of the leakage of CNS molecules through the BBB, blood represents a reliable source of several biomarkers, such as NfL [27]. Other biofluids have been tested, such as urine, from which Simmons et al., 2021 [37] could detect higher p75NTR in HD mice compared to controls. Saliva was demonstrated to contain detectable levels of tHTT, correlating with some HD clinical measures [38] and feces was used to detect bacterial alterations related to clinical features [39]. Finally, hair cortisol content and hair morphology were also hypothesized to be potential prognostic and predictive biomarkers [39,40]. Figure created in BioRender.com.

Thus, taking this into account, many studies are aiming to find biomarkers in more easily accessible biofluids, such as blood, urine or saliva (Figure 1).

#### 3.2.2. Neurofilament Light Protein

One common feature of several neurodegenerative diseases is the impairment of the Blood Brain Barrier (BBB). The compromised BBB, as the disease progresses, leads to the leakage of molecules from the CNS into the blood circulation, which could provide a valid source of biomarker candidates. Indeed, their presence in the bloodstream eases accessibility and collection (compared to CSF), while maintaining high specificity since directly originating from the CNS. One of the recently identified biomarkers that follows this logic is the Neurofilament light protein (NfL, also known as NF-L). The NfL is the smallest of three subunits composing neurofilaments, major components of the neuronal cytoskeleton. NfL is released from damaged neurons and can be detected in blood plasma or serum. Its potential as a prognostic biomarker was first revealed in a retrospective study, conducted by L. M. Byrne and colleagues in 2017 [27]. The study involved the 366 participants of the TRACK-HD cohort, who had been assessed by standardized blood sampling, clinical testing and MRI annually over 3 years. It was observed that NfL concentrations in plasma were significantly higher in all disease stage subgroups among *HTT* mutation carriers compared to the control group. Importantly, the correlation between plasma and CSF NfL concentrations was strong, implying that plasma NfL was indeed CNS-derived and did not originate from other tissues. A correlation was also found between plasma NfL and age in controls and all HD subgroups. In HD patients, NfL concentrations in plasma were positively correlated with cognitive dysfunctions and negatively with the MRI measurements of brain volume (higher NfL values were associated with smaller caudate/putamen volumes) [27,41]. However, whether plasma NfL is able to rapidly change in response to treatment is still not known. For instance, three months were required for serum NfL concentration to normalize after a boxing bout [42]. NfL has undoubtedly proven to be a valuable prognostic biomarker, but the fact that it is released, and subsequently detected, upon neuronal death, classifies NfL as a biomarker for neuronal degeneration, rather than a specific biomarker to track HD progression since prodromal phases.

### 3.3. Other Biomarkers from Accessible Biofluids

In search of other sources of accessible biofluids, several studies started to explore the presence of possible biomarkers in the urine of R6/2 HD mouse models. Indeed, the levels of p75^NTR^ were increased in R6/2 mice compared to WT mice urinary levels [37]. p75^NTR^ was already shown to play a role in memory dysfunction in HD patients [43] and to be altered in HD patients’ striatum [44]. Since urinary p75^NTR^ was shown to be a promising biomarker also for Amyotrophic Lateral Sclerosis [45], this molecule might again represent a more general neurodegenerative biomarker rather than an HD specific one.

The very easily accessible biofluid, saliva (Figure 1) from HD patients, was also tested as a potential source of biomarkers. Here, total huntingtin levels (tHTT) were significantly increased in HD patients compared to healthy controls, correlating with age and several clinical measures [38], supporting the conclusion that salivary tHTT could be a promising non-invasive HD biomarker. HD patients’ saliva, as well as patients’ hair (Figure 1), were also used to detect cortisol levels, with hair cortisol being significantly associated with HD mutation in premanifest individuals [46]. Accordingly, hypocortisolism was already associated with early stages HD patients, possibly due to Hypothalamic–Pituitary–Adrenal (HPA) axis dysfunction [47]. In support of hair as a possible non-invasive source of biomarkers, studies on R6/1 and R6/2 HD mouse models revealed significantly different hair morphology compared to WT mice [40]. This might be of particular interest since, in other metabolic disorders such as Mucopolysaccharidoses (MPS), hair dysmorphology could be identified and then recovered after enzyme-based therapy [48], possibly revealing an easily-available predictive biomarker.

Also, feces might reveal a reliable and non-invasive source of biomarkers (Figure 1). Indeed, 16s RNA sequencing demonstrated that HD patients present an altered gut microbiota, and bacteria diversity correlates with cognitive and clinical measures [39].

Finally, because of its close proximity to the brain, studies testing ocular fluids (e.g., tear fluid) to search for non-invasive biomarkers have been recently carried out for other neurodegenerative disorders such as Alzheimer’s disease [49] and Parkinson’s disease [50], and this might also provide important insights for HD.

## 4. RNA: A New Potential Class of Biomarkers

RNA metabolism dysregulation is a common feature of most neurodegenerative diseases [51]. Accordingly, dysregulated gene expression in HD patients’ brain samples or in in vivo and in in vitro models, has been described, exploiting different genome-wide techniques [52,53,54,55,56,57]. Alteration in RNA expression and processing might be identifiable from the very early stages of the disease, when neurons might be still viable, although not properly functional. For this reason, the interest in RNA biomarkers for diagnostic and prognostic purposes is becoming prevalent.

Several high-throughput studies have highlighted dramatic mRNA changes in the blood of HD individuals [58,59,60,61], leading to the identification of several potential mRNA biomarkers, that not only correlate with the disease stage, distinguishing pre-symptomatic from symptomatic patients [58], but also show association with motor score performances [60]. Importantly, global blood gene expression mirrored the transcriptional dysregulation distinctive of the affected brain areas [61], supporting the correlation between brain and blood transcriptome and the feasibility of using RNA blood biomarkers for brain disorders. Moreover, other low throughput techniques, such as RT-qPCR, were successfully employed to validate some of the potential blood biomarkers, such as the sarco-endoplasmic reticulum-associated ATP2A2 calcium pump (*SERCA2*) and vascular endothelial growth factor transcripts (*VEGF*) [62].

**Figure 2:**
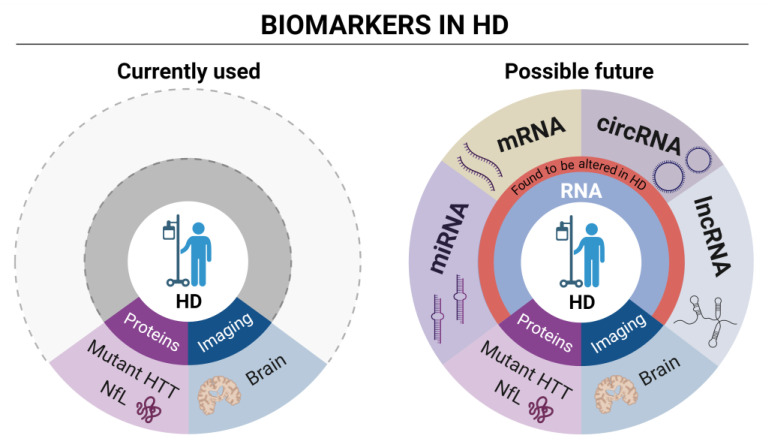
Currently used and possible future RNA biomarkers in HD.

Many RNA species can be found circulating in human biofluids (e.g., blood, urine, saliva, cerebrospinal fluid, breast milk, follicular fluid [63]): these nucleic acids can be broadly categorized as extracellular RNAs (exRNAs), a heterogeneous group of RNAs that includes small microRNAs (miRNAs), long non-coding RNA (lncRNAs), protein-coding RNAs and ribosomal RNAs (rRNAs) (Figure 2). These RNAs can be secreted from cells either in a free form or bound to proteins, as well as in association with extracellular vesicles (EVs) [64]. EVs comprise a variety of membrane-limited vesicles (apoptotic bodies, microvesicles and exosomes) released from cells. Their content, or cargo, consists of lipids, proteins (those associated with the plasma membrane or in the cytosol) and the aforementioned nucleic acids. Indeed, the involvement of EVs in neurodegenerative disorders has been investigated in several studies, showing that EVs have the ability to carry misfolded proteins (e.g., Aβ, α-synuclein, tau) associated with the disease [65,66]. Exosomes, the best characterized subtype of EVs, have crucial roles in normal and pathologic processes and also as possible carriers of biomarkers for diagnostic purposes in clinical settings [67,68]. Several protocols have been already established to execute this from blood serum or urine [69], and even from plasma of HD patients [70].

The cartoon on the left schematically describes the currently used wet (mutant HTT and NfL) protein biomarkers and imaging analysis in HD. The cartoon on the right proposes, instead, focus on different types of RNA biomarkers currently under evaluation in HD clinical research. Differentially expressed genes (DEGs) analysis through high throughput techniques is the most prominent source of mRNA biomarkers, providing hundreds of dysregulated genes as possible HD biomarkers. LncRNAs have been observed to be dysregulated in HD patients. Among them, *DGCR5* was detected, a known interactor of transcriptional repressor REST [71]. Several miRNAs are dysregulated in HD as well (see also Table 1), with some of them, such as miR-10b-5p and miR486-5p representing potential new biomarkers correlating with the CAG tract expansion [72,73]. Finally, circRNAs also represent a new category of promising RNA biomarkers. A recent study detected 23 dysregulated circRNAs in a murine cell line, having functions associated with the dopaminergic synapse [74], while our work, shows dysregulation of circRNAs in neuronal progenitors expressing aberrantly long *Htt* CAG repeat [75]. CircRNA detection and dysregulation in the human blood samples from HD patients remains a still unexplored research area. Figure created in BioRender.com.

If only the 2% of the human transcriptome effectively synthesizes for proteins, the vast majority of the remaining RNAs are indeed ‘non-coding’ (ncRNAs) [76]. ncRNAs, usually shorter and less complex compared to their protein coding counterparts, show a tissue-specific expression pattern and are involved in several crucial cellular processes, such as regulation of transcription, translation and chromatin modulation [77], and have been associated with many neurodegenerative diseases, including HD [78,79]. Then, can ncRNAs function as effective disease biomarkers?

### 4.1. Micro RNA

miRNAs, small ncRNAs responsible for the negative regulation of the expression of genes in a sequence-specific manner by binding to the 3′UTR, promote either the cleavage or translational repression of their target [80,81]. They are involved in a wide range of cellular processes including cell differentiation, metabolism and transcriptional regulation [82], and consequently, alterations in their expression may lead to or influence disease-related pathological phenotypes. In the CNS, miRNAs are abundant, as brain-specific miRNAs contribute to various neuronal processes such as synaptic development, maturation and plasticity [83,84]. In HD, the dysregulation of miRNAs has been extensively reported in in vitro models, transgenic animals and human post mortem brains [72], and thus miRNAs could be included in the list of potential HD biomarker candidates, prompting more detailed investigations. Initial studies by Gaughwin et al. (2011) [85], where *mHtt-Exon-1*-overexpressing human teratocarcinoma cell lines were profiled by microarray, suggested that two miRNAs, miR-34b and miR-1285, were upregulated by mutant huntingtin expression. Subsequent analyses on plasma of HD patients indeed confirmed that miR-34b levels were significantly upregulated in pre-symptomatic HD subjects when compared to controls. Later investigations by Andrew G. Hoss and colleagues in 2015 [73] revealed a group of 75 miRNA, previously identified in *post mortem* brains as significantly altered in HD, also coherently discernible in peripheral blood. As a result, two candidate miRNAs, miR-10b-5p and miR-486-5p, with increasing expression in both HD brain and blood, presented a strong correlation with CAG repeat expansion. In another work, six miRNAs (miR-135b-3p, miR-140-5p, miR-520f-3p, miR-3928-5p, miR-4317, miR-8082) were detected in the CSF as being significantly more expressed in prodromal HD gene carriers than in control, and further increased in patients manifesting the disease [86]. In 2016, Diez-Planelles et al. suggested that the profile of circulating miRNAs might be altered with the progression of the disease [87]. This study, conducted on a group 15 patients (40–45 CAG repeats) and 7 controls, highlighted an inverse correlation between UHDRS total motor score (TMS) and significantly altered levels of miR-122-5p, as such, the lower the TMS, the higher the miR-122-5p expression would be. Coherently, the total functional capacity of HD patients was also associated with reduced levels of miR-330-3p and miR-641. See also Table 1 for general summary.

**Table 1:** Several dysregulated RNAs in Huntington’s Disease.

RNA	Type	Expression Levels in HD	Model System	Reference
miR-34b	miRNA	Up-regulated	mHtt-Exon-1-overexpressing human teratocarcinoma cell lines	[85]
miR-10b-5p, miR-486-5p	miRNA	Up-regulated	*Post mortem* human brains	[73]
miR-135b-3p, miR-140-5p, miR-520f-3p, miR-3928-5p, miR-4317, miR-8082	miRNA	Up-regulated	Human CSF	[86]
miR-122-5p, miR-330-3p, miR-641	miRNA	Up-regulated	Human blood	[87]
*DGCR5*	lncRNA	Down-regulated	Human brain	[71]
*HAR1F, HAR1R*	lncRNA	Down-regulated	Human brain cortex/striatum	[71]
*HttAS_v*	lncRNA	Down-regulated	HEK293, SH-SY5Y	[88]
*NEAT1*	lncRNA	Up-regulated	R6/2 mouse	[89]
*Abhd11os*	lncRNA	Down-regulated	BACHD mouse	[90]
*Meg3*, *Neat1*, *Xist*	lncRNA	Up-regulated	R6/2 mouse	[91]
23 circRNAs	circRNA	dysregulated	PC12 cell line expressing *Htt* exon 1	[74]
>500 cirRNAs	circRNA	dysregulated	mESCs and mNPCs derived from *Htt* mouse models	[75]

### 4.2. Long Non-Coding RNA

LncRNAs are abundant RNAs longer than 200 nucleotides, found to be particularly expressed during embryonic stem cell development and in the brain, involved in several cellular functions, such as transcriptional and chromatin regulation [92]. Several lncRNAs have been found to be dysregulated in HD [71]. Among them, *DGCR5* interacts with transcriptional repressor REST [93], which has been extensively associated with molecular progression of HD [94]. Similarly, two lncRNAs called *HAR1F* and *HAR1R* were detected to be significantly decreased in HD striatum. These lncRNAs are antisense transcripts of the *HAR1* gene, with promoter binding sites for REST [95]. At the *Htt* locus, *HttAS_v,* a lncRNA transcribed as antisense from exons 1 and 3 of the *Htt* gene, was identified [88]. Interestingly, the CAG-expansion mutation causes a downregulation of this lncRNA in the cortex of HD patients, which in turn determines an upregulation of mutant *Htt* expression. Additionally, *NEAT1*, another lncRNA involved in the assembly of nuclear paraspeckles, was observed to be increased in the brain of R6/2 mouse models and HD patients. According to the study, *NEAT1* up-regulation contributes to neuroprotective processes in the presence of the CAG expansion mutation [89]. In the work of Francelle et al., 2015 [90], *Abhd11os* lncRNA was observed to be particularly enriched in the mouse striatum, but downregulated in different HD mouse models. Overexpressing such lncRNA leads to neuroprotective effects, supporting the role of this molecule in the HD striatal vulnerability. Finally, Chanda and colleagues [91], through small RNA-seq performed on R6/2 mouse model brain, detected several dysregulated lncRNAs: among them *Meg3*, *Neat1* and *Xist* showed significant upregulation. Moreover, the knock-down of *Meg3* and *Neat1* reduced the formation of mutant huntingtin aggregates in cell lines over-expressing N-terminal *HTT*. Although still somewhat not always consistent across different model systems and HD patient cohorts, these considerations suggest that monitoring lncRNA levels might represent a new valuable tool to monitor/follow disease progression. See also Table 1 for general summary.

### 4.3. Circular RNA

Circular RNAs (circRNAs) are a subclass of lncRNAs, whose main distinguishing characteristic is their covalently bound 3′ and 5′ extremities, producing their typical single-stranded, closed, circular structure. This implies the lack of any type of terminal modifications, such as the 5′ cap and poly-A tail at the 3′ end [96,97,98,99,100]. Like other RNAs, circRNAs show tissue-specific and/or developmental stage-specific expression patterns, with a peculiar, significant enrichment for the brain districts and higher expression in aging [101]. CircRNAs are generated through the process of backsplicing, where a downstream exon loops back to join a more upstream exon, producing a circularized RNA molecules [99,101,102]. Usually, circularized exons do not retain introns, although this might happen in some cases [103]. Depending on their sequence characteristics and cellular localization, circRNAs have a variety of functions: nuclear circRNAs’ main activities include the modulation of alternative splicing or transcription, regulating the expression of parental genes, interacting with RNA-binding proteins (RBPs) and modulating their activity; in the cytoplasm, instead, they are mainly engaged in sponging miRNAs, thus inhibiting their ability to repress the translation of mRNA [97,98,99,100]. Finally, since a subset of circRNAs possess the translational start codon or an internal ribosome entry site (IRES), it has been observed that they can function as templates for protein synthesis [104]. CircRNAs also exhibit good accessibility, since they have been detected in many types of extracellular body fluids, such as saliva, blood and urine [101,105]. As more of their functions are being elucidated, their underlying relationship with various diseases is being rapidly discovered. Their high stability, specificity and conservation in different tissues add a further dimension to the discovery of these molecules as possible disease biomarkers. Currently available reports clearly show that alterations in the expression of circRNAs play important roles in the development of various pathological conditions, and their potential as biomarkers in neural pathologies is even greater, since their expression is relatively more abundant in the CNS when compared to other tissues [105]. Recently, 23 circRNAs were found to be dysregulated in an HD murine model, overexpressing mutant huntingtin fragment. Their function was associated with dopaminergic synapses, MAPK and long-term depression, all of which were previously related to HD pathogenesis [74]. On the other hand, our genome-wide analyses using mouse knock-in neuronal progenitors series clearly demonstrated a reduction in more than 500 circRNAs following the expression of the expanded CAG alleles [75]. See also Table 1 for general summary. For these reasons, combined with their stability and expression in the blood, circRNAs are emerging as a novel type of disease biomarker that warrants further investigation.

## 5. Conclusions

When dealing with neuro-pathologies such as Huntington’s Disease, it is of utmost importance to be able to enact treatments as soon as possible, optimistically, already during the prodromal stage of the disease. This is not only important to delay the onset of the disease, but also to have the time to personalize treatments to the features and needs of the patients. In order to verify the efficacy of such treatments and to identify the most suitable timeframe for administration, there is a need to develop biomarkers with availability, accessibility, and specificity. The RNA molecules that we have described in this review are one side of the totality of molecules currently being investigated for this purpose, and while some of them are promising, more in-depth investigation, characterization and validation are needed. Finally, once the biomarkers have been chosen, a standardized and reproducible assay for testing needs to be set up, so that all patients may receive their treatment in the appropriate timeframe. Importantly, accessible biomarkers will reduce the suffering to which patients are subjected for their periodic check-ups in the following progression of the disease. Given the severity of HD, it will be important for them to develop less invasive approaches to classify their disease stage or to monitor their response to therapeutic treatments.

## Data Availability

Not applicable.
